# Impact of preeclampsia on cardiovascular events: An analysis of the Generation Scotland: Scottish family health study

**DOI:** 10.1038/s41371-023-00812-2

**Published:** 2023-03-27

**Authors:** Catriona E. Brown, Helen Casey, Anna F. Dominiczak, Shona Kerr, Archie Campbell, Christian Delles

**Affiliations:** 1 School of Cardiovascular and Metabolic Health, University of Glasgow, Glasgow, Scotland UK; 2 MRC Human Genetics Unit, Institute of Genetics and Cancer, University of Edinburgh, Edinburgh, Scotland UK; 3 Centre for Genomic and Experimental Medicine, Institute of Genetics and Cancer, University of Edinburgh, Edinburgh, Scotland UK

**Keywords:** Medical research, Risk factors

## Abstract

Preeclampsia is a recognised cause of an increased risk of major adverse cardiovascular events when compared to the background risk in women who did not have hypertensive disorders during pregnancy. The Generation Scotland: Scottish Family Health Study (GS:SFHS) is a population cohort of more than 20,000 members of the Scottish population. Using the Scottish Morbidity Records, we linked the women in the GS:SFHS cohort to validated maternity and inpatient admission data. This allowed us to robustly identify cardiovascular outcomes in the form of inpatient admission for cardiovascular events, We also aimed to explore the risk of pregnancy on future cardiovascular events, using data from nulliparous and parous women.In total, 9732 women were selected. 3693 women were nulliparous, and after study exclusion, 5253 women with 9583 pregnancies remained. Pregnancies from 1980 until the end of the study period of 1st of July 2013 were included. Cardiovascular events occurred in 9.0% of nulliparous women, 4.2% of women with pregnancies and in 7.6% of women with a history of preeclampsia. A total of 218 parous women experienced cardiovascular events, 25 in the preeclampsia group and 193 in the normotensive group.Survival analysis was undertaken, with index pregnancy taken as first pregnancy in normotensive controls and first preeclampsia pregnancy in cases. Endpoint of interest was admission to hospital with first cardiovascular event. After further exclusions a total of 169 cardiovascular events occurred in the normotensive pregnancy group and 20 in the preeclampsia group. Women with a history of preeclampsia were more likely to have cardiovascular events later in life than women with normotensive deliveries., This was statistically significantly different on Kaplan Meier survival analysis, (log rank Mantel-Cox *p*-value < 0.001). The women in our study were middle-aged, within 33 years of pregnancy, with a mean age of 53 years in the preeclampsia cardiovascular events group.Our study supports the urgent need for uniform guidelines and implementation to improve the health in women with this medical history. Increased awareness among the public of the cardiovascular risk associated with PE is vital to aid uptake of cardiovascular prevention programmes.

## Introduction

Pre-eclampsia (PE) is a leading cause of maternal and neonatal morbidity and mortality affecting 2–5% of all pregnancies [1]. It is now well recognised worldwide that women with a history of preeclampsia have approximately 1.2–3 times increased risk of major adverse cardiovascular events when compared to the background risk in women who did not have hypertensive disorders during pregnancy [2–6].

A large Norwegian cohort with 626,272 births from 1967 to 1992 with a follow up of up to 25 years (median 13 years) showed that women who had preeclampsia had a 1.2-fold higher long-term risk of cardiovascular disease (CVD) mortality (CI 1.02 to 1.37) than those without preeclampsia [3]. Using the Child Health and Development Studies Pregnancy Cohort in San Francisco, 14,062 women were investigated for the relationship between pregnancy complications and CVD mortality from time of enrolment in the 1960s to 50 years subsequent follow up. Preeclampsia was the pregnancy complication associated with highest risk of CVD mortality in this cohort; early preeclampsia (HR 3.6 CI 1.04–12.19) was associated with a higher risk than late preeclampsia (HR 2.0 CI 1.18–3.46) [5].

The ongoing population-based cohort study Nord-Trøndelag Health (HUNT) Study is of all residents in Norway’s Nord-Trøndelag County over 20 years of age, where every decade they are invited to participate in an extensive health assessment, including a clinical examination and questionnaires. This cohort has been investigated for association between pregnancy complications and cardiovascular morbidity and mortality [4]. A large study linked the HUNT data to the Medical Birth Registry of Norway validated hospital records, and the Norwegian Cause of Death Registry. This allowed 18,231 women to be investigated for CVD end points of interest: non-fatal myocardial infarction; fatal myocardial infarction; non-fatal stroke; and fatal stroke [4]. Preeclampsia was found to be the only pregnancy complication that predicted an increased risk of CVD after adjusting for established risk factors (HR 1.60, 95% CI 1.16–2.17) [4]. The study however excluded women under 40 at the time of follow-up, and evidence exists that the association of pregnancy complications with CVD is stronger at younger ages [7].

The Generation Scotland: Scottish Family Health Study (GS:SFHS) is another general population cohort and consists of questionnaire data, clinical data and biosamples of more than 20,000 members of the Scottish population [8]. Study participants were recruited in Clinical Research Facilities located in the cities of Aberdeen, Dundee, and Glasgow between 2006-2011 [8]. GS:SFHS did not collect maternity data at the time of recruitment, and has therefore not been used to explore pregnancy-related outcomes. By using the Scottish Morbidity and Mortality Records, we aimed to link the women in the GS:SFHS cohort to validated maternity and inpatient admission data. This has allowed us to explore inpatient admissions in relation to maternity data in this cohort.

This study sought to investigate the association between preeclampsia and future cardiovascular events in women who had volunteered to join GS:SFHS. We hypothesised nulliparity may be a significant risk factor for future cardiovascular disease in women. We therefore also aimed to explore the risk of cardiovascular events in nulliparous and parous women.

## Subjects and methods

### Data linkage

For the purpose of this study, the participants of the GS:SFHS cohort were linked to their NHS data in the Scottish Morbidity Records (SMR). Information Services Division (ISD) NHS Scotland hold a data catalogue of NHS health and health-related data for the population of Scotland in the SMR. SMR datasets used in this study were SMR01 “General/Acute inpatient and Day Case” and SMR02 “Maternity Inpatient and Day Case”. These datasets hold clinical data on hospital admissions and obstetric history which are coded according to the WHO’s International Classification of Disease (ICD) coding system. In this study ICD-9 and ICD-10 classifications were used. The ISD have assessed the quality of SMR01 and SMR02 data, and data collection has been > 99% complete since the 1970s. Record linkage was facilitated by GS:SFHS team, ISD NHS Scotland and the Health Informatics Centre, Dundee. Each participant had SMR01 data and SMR02 data linked and pseudonymised by a new unique identifier, before the data was passed securely to the research team [9].

SMR01 data for cardiovascular events were only included if listed as the main condition for hospital admission. Cardiovascular events were taken to include ICD-9 and ICD-10 coding for rheumatic heart disease, hypertensive diseases, pulmonary heart diseases, any other forms of heart disease, cerebrovascular diseases, diseases of the arteries, arterioles and capillaries, and other circulatory diseases. Disorders of the venous system were excluded.

Pregnancies from 1980 until the end of the study 1st of July 2013 for SMR02 data were included. SMR02 data classified as a case of preeclampsia as per ICD-9 codes or ICD-10 codes were included. Those pregnancies coded with hypertensive disorders of pregnancy but not preeclampsia were excluded.

Generation Scotland data on age at recruitment, anthropometric measurements, clinical measurements (blood pressure and heart rate), biochemistry and self-reported clinical diagnosis (hypertension, diabetes and smoking history) were used for analysis.

### Study cohort

**Fig. 1:**
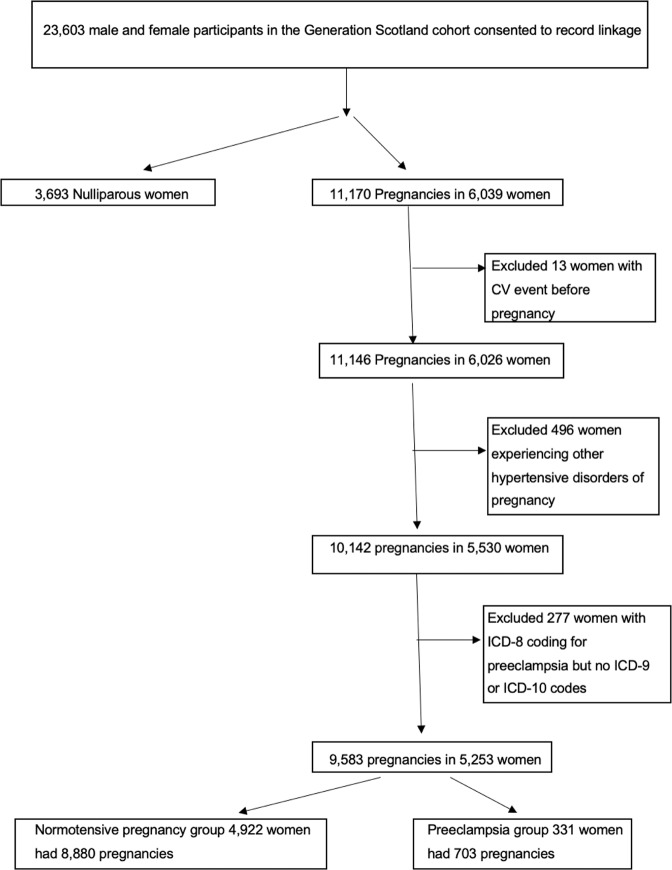
Identification of women with preeclampsia in the Generation Scotland: Scottish Family Health Study (GS:SFHS).

A flow diagram in Fig. 1 demonstrates the identification of preeclampsia cases and normotensive controls within GS:SFHS. There were 9,732 women with SMR01 and SMR02 data available. 3693 women were nulliparous, and 6039 women had 11,170 pregnancies. Thirteen experienced a cardiovascular event before their first pregnancy, a further 496 women had experienced other hypertensive disorders of pregnancy and were therefore excluded. 277 were excluded as they had less reliable ICD-8 coding for pre-eclampsia and no ICD-9 or ICD-10 codes. A total of 5253 women with 9583 pregnancies remained. 4922 women had 8880 normotensive pregnancies and 331 women with 703 pregnancies were in the pre eclamptic group. Out of these 331 women, there were 359 actual preeclamptic pregnancies. We were unable to access information about whether these preeclamptic pregnancies were classified as early or late preeclampsia as this information was not recorded.

### Statistical Analysis

The dataset was initially prepared using R (http://R-project.org), removing duplicates and merging SMR01 and SMR02 and homogenisation of ICD codes. Statistical analysis was performed using IBM SPSS statistics for Windows (Version 22.0, Armonk, NY: IBM Corp 2013). For comparison of continuous variables independent samples t-test or Mann-Whitney U test were used depending on the distribution of the data. Categorical variables were compared using Chi-squared test. Continuous variables were summarised using mean ± standard deviation or medians and interquartile range, depending on data distribution. Survival analysis was carried out with the endpoint “first cardiovascular event”. Index pregnancy was defined as “first pregnancy” in normotensive controls and “first preeclamptic pregnancy” in cases. Correction was performed for; multiple pregnancies, low birth weight (<2500 g), birth at gestation <37 weeks.

## Results

### Baseline data

In the pre eclamptic group, preeclampsia occurred in the first pregnancy in 237/331 (71.6%) women, the second pregnancy in 76/331 (23%) women and the third pregnancy in 18/331 (5.4%) women. A total of 303/331 (91.5%) women experienced preeclampsia in only one pregnancy and 28/331 (8.5%) experienced preeclampsia in two pregnancies.

The index pregnancy was defined as the first pregnancy in normotensive women and the first preeclamptic pregnancy in those with preeclampsia. None of the pregnancies had gestations < 20 weeks. There was no difference in age at index pregnancy between the normotensive and preeclamptic group (*p* = 0.389). On adjusting birth weight for gestational age using Z scores, there was no statistically significant difference between the two groups (*p* = 0.456). Women with a diagnosis of preeclampsia were more likely to have a caesarean section at delivery (*p* <0.001) and gave birth at an earlier gestation (*p* <0.001).

At the time of recruitment to GS:SFHS, women with preeclampsia had a significantly higher body mass index (*p* = <0.001), systolic blood pressure (*p* <0.001) diastolic blood pressure (*p* <0.001), heart rate (*p* = <0.001), current diagnosis of hypertension (*p* <0.001) and diabetes (*p* <0.001) than the normotensive pregnancy group. Nulliparous women (*N* = 3693) had a significantly higher body mass index (*p* = 0.015), systolic blood pressure (*p* <0.001) and a higher proportion of hypertension (*p* <0.001) and diabetes (*p* <0.001) than parous women (*N* = 5253). Parous women were more likely to smoke at time of recruitment than nulliparous women (*p* = 0.002) (Table 1).

**Table 1:** Characteristics of the cohort at the time of the study visit.

Measurement	Nulliparous (*N* = 3693)	Parous (*N* = 5253)	*p*-value	Age-adjusted *p*-value
Age (yrs)	49.4 ± 18.1	46.5 ± 10.9	**<0.001**	N/a
Height (cm)	161.9 ± 6.8	162.6 ± 6.3	**<0.001**	**0.032**
Weight (kg)	70.2 ± 15.4	69.7 ± 14.7	0.0182	0.139
BMI (kg/m^2^)	26.8 ± 5.8	26.3 ± 5.4	**0.001**	**0.015**
Mean SBP (mmHg)	130.9 ± 19.3	125.6 ± 17.1	**<0.001**	**<0.001**
Mean DBP (mmHg)	78.5 ± 10.1	77.8 ± 10.0	**0.004**	0.608
Mean HR (bpm)	71.0 ± 11.1	70.4 ± 10.7	**0.009**	**0.001**
Glucose (mmol/L	4.8 ± 1.0	4.7 ± 1.0	**<0.001**	**0.007**
Total cholesterol (mmol/L)	5.1 ± 1.1	5.2 ± 1.1	**<0.001**	**<0.001**
HDL cholesterol (mmol/L)	1.583 ± 0.414	1.582 ± 0.410	0.89	0.175
Sodium (mmol/L)	139.6 ± 2.5	139.8 ± 2.3	**0.003**	**<0.001**
Potassium (mmol/L)	4.17 ± 0.46	4.13 ± 0.41	**<0.001**	**<0.001**
Urea (mmol/L)	5.0 ± 1.6	4.8 ± 1.2	**<0.001**	**<0.001**
Creatinine (μmol/L)	67.9 ± 12.3	66.4 ± 10.4	**<0.001**	**<0.001**
Current hypertension	606/3637 (16.7%)	492/5143 (9.6%)	**<0.001**	
Current diabetes	123/3637 (3.4%)	72/5143 (1.4%)	**<0.001**	
Current smoker	563/3583 (15.7%)	933/5100 (18.3%)	**0.002**	

The table displays the comparison of clinical and biochemical measurements taken at time of recruitment to GS-SFHS in remaining 8946 women following exclusion of ineligible participants.

*SBP* Systolic blood pressure, *DBP* Diastolic blood pressure,

*HR* Heart rate, *HDL* High density lipoprotein, *BMI* Body Mass Index.

*Data are displayed as mean ± standard deviation

Statistically significant *p*-values are in bold.

### Cardiovascular events

In the 333 nulliparous women with cardiovascular events, there were 909 separate cardiovascular admissions. Of the 218 parous women with cardiovascular events, there were 634 separate hospital admissions. In the parous subgroup with preeclampsia, 25 women had cardiovascular events with 57 hospital admissions (Table 2). Cardiovascular events occurred in 9% of nulliparous women, 4.2% of women with pregnancies and in 7.6% of women with a history of preeclampsia. There is a statistically significant difference with more cardiovascular events in nulliparous than parous women (*p* <0.001).

**Table 2:** Comparison of cardiovascular event hospital admission types in parous vs parous women with preeclampsia.

Hospital Admission Type	218 Parous women with 634 cardiovascular events	25 Parous women with a history of preeclampsia with 57 cardiovascular events
Rheumatic heart disease	1%	0%
Ischaemic heart disease	31%	19%
Other forms of heart disease	21%	37%
Hypertensive diseases	3%	14%
Pulmonary heart disease	5%	3%
Cerebrovascular disease	13%	18%
Diseases of the arteries and capillaries	25%	9%
Other unspecified circulatory disorders	1%	0%

Of the 218 women who had experienced cardiovascular events since pregnancy, 25 were in the preeclampsia group and 193 in the normotensive group. There were no differences in the anthropometric or biochemical measurements in those with preeclampsia versus those with normotensive pregnancy, except for a significantly higher serum creatinine in those with history of preeclampsia (*p* = 0.018) (Table 3).

**Table 3:** Descriptive results for preeclampsia cases and normotensive controls in 5253 women by cardiovascular event vs no cardiovascular event.

	Cardiovascular event (*N* = 218)			No Cardiovascular event (*N* = 5035)		
Measurement	Normotensive Pregnancy (*N* = 193)	Preeclampsia	*p*-value	Normotensive Pregnancy	Preeclampsia	*p*-value
		(*N* = 25)		(*N* = 4729)	(*N* = 306)	
Age (yrs)	54 (10)	53(9)	0.578	47 (17)	49 (12)	**0.01**
Height (cm)	161.2 ± 6.0	160.0 ± 6.5	0.326	163.0 (8.2)	162.0 (9.9)	0.135
Weight (kg)	70.7 (23.5)	76.4 (26.0)	0.858	66.7 (17.1)	69.3 (19.7)	**<0.001**
BMI (kg/m^2^)	27.2 (9.7)	27.0 (10.5)	0.645	25.2 (6.4)	26.6 (7.1)	**<0.001**
Mean SBP (mmHg)	132 (30)	135 (22)	0.445	122 (21)	130 (23)	**<0.001**
Mean DBP (mmHg)	79 (15)	81 (12)	0.283	77 (12)	82 (12)	**<0.001**
Mean HR (bpm)	70 (12)	73 (11)	0.225	69 (14)	72 (14)	**<0.001**
Glucose (mmol/L	4.7 (0.7)	4.8 (1.8)	0.243	4.6 (0.5)	4.6 (0.7)	**0.007**
Total cholesterol (mmol/L)	5.2 ± 1.2	5.1 ± 1.1	0.694	5.1(1.4)	5.2 (1.3)	0.425
HDL cholesterol (mmol/L)	1.5 ± 0.4	1.5 ± 0.4	0.675	1.5 (0.5)	1.5 (0.5)	0.564
Sodium (mmol/L)	140 (4)	140 (2)	0.317	140 (3)	140 (2)	0.109
Potassium (mmol/L)	4.1 (0.5)	4.1 (0.6)	0.465	4.1 (0.4)	4.1 (0.4)	0.692
Urea (mmol/L)	5.1 (1.7)	5.1 (1.2)	0.932	4.6 (1.6)	4.8 (1.5)	0.204
Creatinine (μmol/L)	65 (12)	73 (23)	**0.018**	65 (13)	66 (12)	0.188
Current hypertension	42/187 (25.1%)	13/25 (52%)	**0.005**	351/4631 (7.6%)	81/300 (27%)	**<0.001**
Current diabetes	10/187 (5.3%)	5/25 (20%)	**0.007**	49/4631 (1.1%)	8/300 (2.7%)	**0.012**
Current smoker	46/186 (24.7%)	7/25(28%)	0.723	840/4591 (18.3%)	40/298 (13.4%)	**0.034**

*SBP* Systolic blood pressure, *DBP* Diastolic blood pressure, *HR* Heart rate, *HDL* High density lipoprotein, *BMI* Body Mass Index.

Statistically significant *p*-values are in bold.

In the pre eclamptic group (*N* = 331), there was a significantly higher creatinine in women with a cardiovascular event (*p* = 0.028) and a significantly higher proportion with a diagnosis of hypertension (*p* = 0.008), diabetes (*p* <0.001) and smoking (*p* = 0.047) compared with those who had not had a cardiovascular event. However, women in the cardiovascular event group were statistically significantly older at time of recruitment into GS:SFHS than those who did not experience a cardiovascular event (*p* = 0.008) (Table 4).

**Table 4:** Descriptive results for cardiovascular events vs no cardiovascular events by preeclampsia vs normotensive pregnancy in 5,253 women.

	Preeclampsia (*N* = 331)			Normotensive pregnancy (*N* = 4922)		
Measurement	No CV event (*N* = 306)	CV event (*N* = 25)	*p*-value	No CV event (*N* = 4729)	CV event (*N* = 193)	*p*-value
Age (yrs)	49 (12)	53 (9)	**0.008**	47 (17)	54 (10)	**<0.001**
Height (cm)	162.0 (9.9)	161.0 (8.7)	0.088	163.0 (8.2)	161.0 (8.0)	**0.003**
Weight (kg)	69.3 (19.6)	76.4 (26.0)	0.683	66.7 (17.1)	70.7 (23.5)	**0.001**
BMI (kg/m^2^)	26.6 (7.1)	27.0 (10.5)	0.331	25.2 (6.4)	27.2 (9.7)	**<0.001**
Mean SBP (mmHg)	130 (23)	135 (22)	0.449	122 (21)	132 (30)	**<0.001**
Mean DBP (mmHg)	83 ± 9	81 ± 9	0.3	77 (12)	79 (15)	**0.009**
Mean HR (bpm)	73 ± 11	73 ± 11	0.863	69 (14)	70 (17)	0.458
Glucose (mmol/L	4.6 (0.7)	4,8 (1.8)	0.076	4.6 (0.5)	4.7(0.7)	**<0.001**
Total cholesterol (mmol/L)	5.3 ± 1.1	5.1 ± 1.1	0.347	5.1 (1.4)	5.1 (1.3)	0.264
HDL cholesterol (mmol/L)	1.5 (0.5)	1.5 (0.4)	0.818	1.5 (0.5)	1.5 (0.6)	**0.015**
Sodium (mmol/L)	140 (2)	140 (2)	0.19	140 (3)	140 (4)	0.347
Potassium (mmol/L)	4.1 (0.4)	4.1 (0.6)	0.922	4.1 (0.4)	4.1 (0.5)	**0.028**
Urea (mmol/L)	4.8 (1.5)	5.1 (1.2)	0.143	4.6 (1.6)	5.1 (1.7)	**<0.001**
Creatinine (μmol/L)	66.8 ± 9.4	75.2 ± 17.0	**0.028**	65 (13)	65 (12)	0.416
Current Hypertension	81/300 (27%)	13/25 (52%)	**0.008**	351/4631 (7.6%)	47/187 (25.1%)	**<0.001**
Current Diabetes	8/300 (2.7%)	5/25(20%)	**<0.001**	49/4631 (1.1%)	10/187 (5.3%)	**<0.001**
Current Smoker	40/298 (13.4%)	7/25 (28%)	**0.047**	840/4591 (18.3%)	46/186 (24.7%)	**0.027**

*SBP* Systolic blood pressure, *DBP* Diastolic blood pressure, *HR* Heart rate, *HDL* High density lipoprotein, *BMI* Body Mass Index.

Statistically significant *p*-values are in bold.

In the normotensive pregnancy group (*N* = 4,922), there was a significantly higher body mass index (*p* = 0.001), systolic blood pressure (*p* <0.001), diastolic blood pressure (*p* = 0.009), higher glucose level (*p* <0.001) and larger proportion of current smokers (*p* = 0.027) in those with a cardiovascular event compared to those without any cardiovascular events. The age at time of recruitment was statistically significantly older in the cardiovascular event group at time of recruitment into GS:SFHS than in those who did not experience a cardiovascular event (*p* = <0.001) (Table 4).

**Fig. 2:**
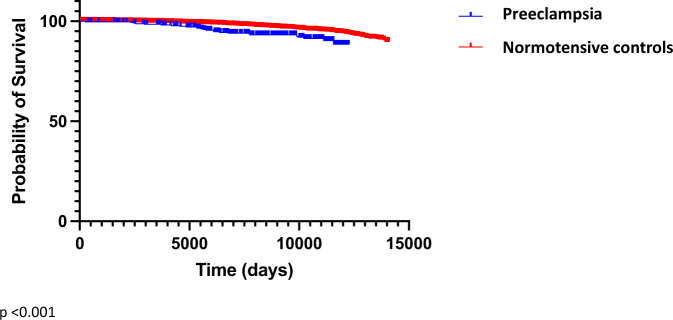
Survival curves displaying time to cardiovascular event in preeclampsia vs normotensive controls. *P*-value derives from log-rank (Mantel-Cox) test.

Survival analysis was undertaken as stated previously with index pregnancy taken as first pregnancy in normotensive controls and first preeclampsia pregnancy in cases. Admission to hospital with first cardiovascular event was the endpoint of interest. In women who did not experience the endpoint of interest, censoring occurred either when the end of the study was reached (1st July 2013) or if they experienced death from a non-cardiovascular admission diagnosis. Correction was performed for possible confounders by removal of 277 women who delivered babies with low birth weight (<2500 g), 107 with pre-term delivery (<37 weeks’ gestation) and 35 with multiple pregnancies. This left 4834 women, 270 with preeclampsia and 4564 with normotensive pregnancy for survival analysis. A total of 169 cardiovascular events occurred in the normotensive pregnancy group and 20 in the preeclampsia group, which was statistically significantly different on Kaplan Meier survival analysis, (log rank Mantel-Cox *p*-value <0.001) (Fig. 2).

## Discussion

We used the data linkage of two well validated datasets to allow us to robustly identify cardiovascular events by inpatient admission. This linkage allowed us to characterise our cohort in detail and analyse their maternity data.

In the GS:SFHS cohort women with a history of preeclampsia had a higher blood pressure and greater risk of cardiovascular events later in life than women with normotensive pregnancies. Women with a history of preeclampsia experienced earlier gestational age at delivery and were more likely to have a caesarean section birth. On survival analysis, women with a history of preeclampsia were more likely to have cardiovascular events later in life than women with normotensive deliveries. The women in our study were within 33 years of pregnancy with a mean age of 53 years in the PE cardiovascular events group. Although our study only had a small number of 25 women who had a cardiovascular event after preeclamptic pregnancy these results are consistent with other studies. A 2017 meta-analysis of 22 studies and >2,500,000 women showed the risk for cardiovascular event was highest 1 to 10 years postpartum, supporting our data that adverse cardiac events may occur before middle age [6, 10]. Close follow up in the years postpartum even in a young cohort with focus on preventative medicine may help to reduce the cardiovascular risk in this group.

Nulliparous women were found to have a greater risk of cardiovascular events than parous women. We were unable to differentiate nulliparous women who chose not to have children from those who could not become pregnant within our cohort. This higher risk associated with nulliparity may therefore reflect the relationship between general health and fertility. The nulliparous group had a significantly higher BMI, blood pressure and higher proportion of hypertension than our parous group. However, our findings are supported by other studies reporting increased cardiovascular risk associated with nulliparity [11, 12]. To examine the impact of parity future studies should try to differentiate nulliparous women who chose to not become pregnant and those who could not become pregnant, as combining these groups may falsely attribute nulliparity to greater cardiovascular risk or mask an even higher risk within subgroups of the nulliparous population.

The strength of this study was in using the ICD classification and SMR01 and SMR02 data. This does not depend on recall of participants and is therefore not subject to this bias. The ISD data undergoes quality assessment, and rigorous exclusion of any pregnancy which was not deemed preeclampsia was carried out. Exclusion of any non-normotensive pregnancy was also applied to the control group. Cardiovascular events were evaluated in nulliparous women in addition to those with preeclampsia and normotensive pregnancies.

A limitation of this study is that only women who had lived long enough to enrol in the Generation Scotland study were included, some women who exhibited cardiovascular events earlier and suffered cardiovascular mortality are therefore not included. The women who were participants in Generation Scotland may have been more interested than average in their own health and general fitness and wellbeing, so therefore may not be fully representative of the Scottish population. Due to sample size, it was not possible to assess the effect of recurrent preeclampsia or to compare women with multiple to single pregnancies. We are aware of different severity and possible prognostic differences between the early and late PE. For our study, differentiating between early and late PE was not possible. These limitations have wider implications on the robustness of our adjustments particularly in survival analysis. Different exposures of cases and controls to risk and disease factors at selection and during follow-up should ideally be taken into account but with our focus on cardiovascular events, time-varying models to take changes and differences in exposure into account were not possible.

Women with a history of PE have been recognised to have an increased risk of long-term cardiovascular events including heart failure, coronary artery disease, and stroke [3–7, 10, 13–15]. The Cardiovascular health after maternal placental syndromes (CHAMPS) population-based cohort study looked at 1.03 million women, free from cardiovascular disease before their first documented delivery, between 1990 and 2004 enrolled in the Ontario Health Insurance Plan, which holds all maternity data [7]. To analyse outcomes, the data was linked to the mortality data from the Canadian Registered Persons Database and to hospital admissions data from Canadian Institute for Health Information Discharge Abstract Database. The primary outcome was a composite of cardio-vascular disease, defined as hospital admission for coronary, cerebrovascular, or peripheral artery disease, or revascularisation of the coronary, carotid, or a lower extremity arterial system [7]. The median duration of follow-up was 8·7 years. The preeclampsia group (*n* = 36982) showed an increased risk of premature cardiovascular disease (HR2·1 CI 1·8–2·4) with an average age of 38 years at their first cardiovascular event [7].

This study gives further evidence to the need for early postpartum and long term follow up for women with a history of PE [6, 10, 13, 15–21]. There is a need for identification of biomarkers with a strong predictive value of future postpartum CVD in women with a history of PE [22]. Increased levels of angiogenic factors such as soluble endoglin and increased ratios of soluble Fms-like tyrosine kinase 1: placental growth factor have been identified as clinically useful predictive and diagnostic biomarkers in the development of preeclampsia however to date no specific markers for future CVD risk has been identified [23]. Biophysical markers such as pulse wave velocity, flow mediated dilatation and carotid intima-media thickness should also be explored as a possible predictive tool of future CVD risk [24]. If biomarkers could be identified then resources could be personalised and targeted towards those most at risk of future CVD. This presents an important avenue for future research in this field.

Currently guidelines around timing of follow up, cardiovascular screening and preventive strategies for cardiovascular disease in the cohort who have history of previous preeclampsia vary worldwide and consensus is lacking. There is an urgent need for uniform guidelines and implementation to improve the health in this cohort. Increased awareness among the public of the cardiovascular risk associated with PE is vital to aid uptake of cardiovascular prevention programmes.

While outside the scope of this study, there is increased recognition of the increased CVD risk experienced by the offspring of women with a history of PE [22, 23, 25–27]. Future research into this important aspect of this topic and guidelines surrounding CVD risk prevention in the offspring of this cohort may also be warranted.

## Summary

### What is known about the topic

- Pre-eclampsia is a leading cause of maternal and neonatal morbidity and mortality affecting 2–5% of all pregnancies.
- It is now well recognised worldwide that women with a history of preeclampsia have approximately 1.2–3 times increased risk of major adverse cardiovascular events when compared to the background risk in women who did not have hypertensive disorders during pregnancy.

### What this study adds

- This study sought to investigate the association between preeclampsia and future cardiovascular events in women who had volunteered to join the Generation Scotland: Scottish Family Health Study (GS:SFHS).
- We hypothesised nulliparity may be a significant risk factor for future cardiovascular disease in women.

## Data Availability

Data will be made available to researchers upon reasonable request and in accordance with terms and conditions set by Generation Scotland. Email access@generationsotland.org for further details.
